# Finite Element Analysis and Computational Framework for Optimizing Laser Surface Modified Ti-6Al-4V Femoral Components in Total Knee Replacement

**DOI:** 10.3390/mi17060740

**Published:** 2026-06-18

**Authors:** Iman Shakir Tawfeeq, Hussam Lefta Alwan, Taha A. Elwi

**Affiliations:** 1 College of Electromechanically Engineering, University of Technology, Baghdad 10066, Iraq; eman.s.towfeeq@uotechnology.edu.iq; 2 College of Production Engineering and Metallurgy, University of Technology, Baghdad 10066, Iraq; hulefta76@yahoo.com; 3 Department of Automation and Artificial Intelligence Engineering, College of Information Engineering, Al-Nahrain University, Baghdad 64074, Iraq

**Keywords:** TKR, Ti-6Al-4V, LSR, FEA, MPD, GUI

## Abstract

Titanium alloys such as Ti-6Al-4V are widely used in orthopedic implants due to their strength and biocompatibility. Laser Surface Remelting (LSR) offers a promising approach to modify surface properties without altering bulk characteristics. This study investigates the effects of varying melt pool depths (MPDs) from 0 μm to 30 μm in 10 μm steps on the mechanical behavior of Ti-6Al-4V femoral components in Total Knee Replacement (TKR) using a comprehensive computational approach combining Finite Element Analysis (FEA) and computational algorithm-based automated evaluation. A three-dimensional FEA model was developed and tested under four physiological loading conditions: compression, axial distraction, medial bending, and lateral flexion at 1300 N. Results show that increasing MPD from 0 μm to 30 μm increases the maximum von Mises stress by 4.2% under compression but reduces displacement by up to 51.7% under distraction. An MPD of 20 μm reduces displacement by 48% while increasing stress by only 2.7%, representing an optimal balance. The computational algorithm framework identifies 15–25 μm as the optimal range for balancing surface enhancement with mechanical integrity. Experimental validation shows good agreement between simulated and measured results, confirming the reliability of the proposed framework for optimizing surface modification parameters in orthopedic implants.

## 1. Introduction

The rapidly growing need for TKRs is coupled with concerns about aseptic loosening, and the longevity of implants [1], which identifies aseptic loosening as the primary failure mode, has augmented the request for innovative technologies aimed at enhancing material performance [2], highlighting the need for improved implant longevity. Titanium alloys, particularly Ti-6Al-4V, are frequently regarded as the optimum options for orthopedic implants [3], due to their corrosion resistance [4], exceptional biocompatibility [5], and favorable strength-to-weight ratio [6]. Given the characteristic bio-inertness of titanium alloys, surface alterations are frequently essential to confirm the long-term functionality of implants and facilitate actual bone incorporation [7].

The usage of LSR has increased importance in surface engineering due to its precision [8], simplicity of control [9], and size to produce adapted surface microstructures without introducing additional materials [10]. The melting process with LSR is very fast and leaves a thin layer on the surface [8] to make the surface much stronger, less likely to wear down and more ordered on a very small scale [11]. The depth of lasing regarding MPDs depends on laser power density, scanning speed, and beam width [12]. The realized surface quality is significantly affected by MPD [12].

For this, different researches have been done on the microstructure and wear properties of Ti-6Al-4V after LSR treatment [3]. However, different MPDs affecting the complete biomechanical performance of complex orthopedic mechanisms have not been studied in depth [11]. For this, varying the LSR settings might involve repeating the same tests. The importance of developing a mathematical model with clear vision has not been stated yet. To report these issues, a comprehensive computational framework is established for this investigation. This framework integrated FEA with automated analytical techniques, all executed within the computational algorithm environment. The primary objectives of this research are threefold: initially, to examine the influence of varying LSR of MPD on the stress distribution and bending properties of Ti-6Al-4V TKR femoral components; subsequently, to construct a comprehensive computational algorithm for parameter optimization and result visualization; and lastly, to formulate design criteria for the optimal LSR parameters over multi-criteria performance calculations. Despite the well-established benefits of LSR for enhancing surface hardness and wear resistance of Ti-6Al-4V, the systematic study of how different MPDs affect the overall biomechanical performance of complex orthopedic implants under physiologically relevant loading conditions is still a crucial research gap. Previous studies have mainly investigated the microstructural and surface properties of flat or simple geometry specimens, ignoring the stress distribution, displacement behavior and multi-criteria performance trade-offs in anatomically contoured femoral components. Moreover, there has been no prior research that combines FEA and an automated computational algorithm-based optimization system specifically developed to determine the optimal LSR parameters for TKR applications. This study addresses these gaps by (1) systematically evaluating MPDs ranging from 0 μm to 30 μm across four physiological loading conditions, (2) developing a comprehensive computational framework that automates parameter optimization, and (3) establishing clinically relevant design criteria for LSR-treated Ti-6Al-4V femoral components.

## 2. Related Work

The use of FEA has been converted to a vital technique for investigating biomechanics for different formulates including knee joints [13]. Scientific communities have applied image-based policies to create realistic knee joint replicas [11], whereas some researchers examined the contact behavior and stress distributions on knee prostheses [1]. Usually, FEA studies approach implant materials as if they are all the same, disregarding the different properties that come from varying the surface [6].

Researchers applied the use of LSR-treated Ti-6Al-4V, where they mainly concentrated on microstructural characterizations [10], improvement of surface hardness [14], and enhancement of wear resistance [14]. In [15], the authors showed that LSR improved surface hardness from 25% to 35% by refining the grains. The researchers in [11] explored that optimal biocompatibility may be realized when MPD is between 20 μm and 25 µm, in contrast to current research, which delivers incomplete information about how property changes caused by LSR impact the complete performance of mechanisms used in biological surroundings.

Current progressions in computational approaches have simplified the optimization of implant projects. Decision-making methods that reflect multiple standards and computational frameworks [16] have been used to select materials and refine designs. The integration of FEA with programming-based MATLAB R2024a environments [17] may offer significant compensation for systematic parameter investigations and automated studies. Therefore, this work presents a novel method that combines FEA with a complete computational algorithm framework to methodically train effects on LSR parameters and make it easier to design implants.

## 3. Methodology

**A.**  **Finite Element Modeling**

**Figure 1:**
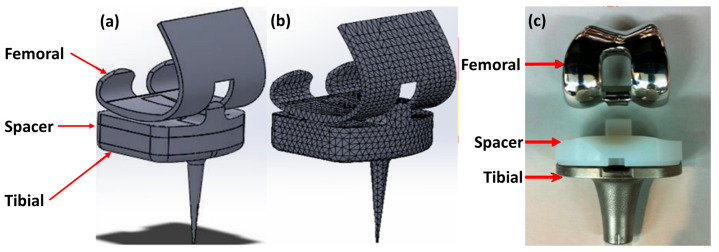
Finite element model of the TKR assembly: (**a**) 3D CAD model showing femoral component, tibial tray, and polyethylene insert; (**b**) tetrahedral mesh with local refinement in high-stress regions; (**c**) isolated view of the Ti-6Al-4V femoral component.

**Figure 2:**
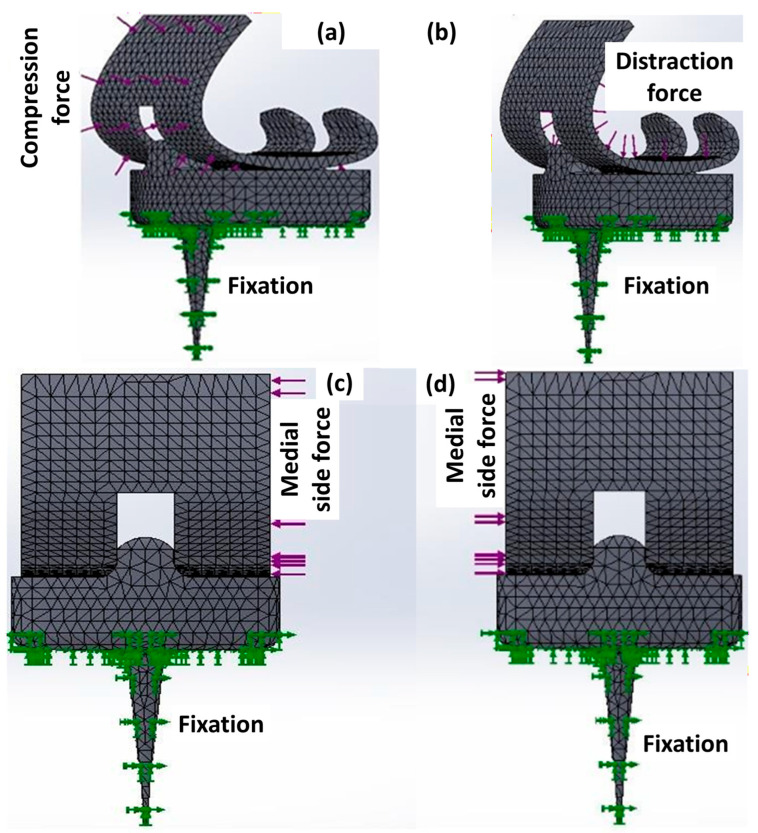
Physiological loading conditions (1300 N) applied to the TKR model: (**a**) axial compression (−Z direction); (**b**) axial distraction (+Z direction); (**c**) medial bending (+X direction); (**d**) lateral bending (−X direction). Color contours represent von Mises stress distribution (blue: minimum, red: maximum).

The considered FEA model of TKR is realized based on 3D assembly, counting the femoral parts, the tibial module, and the polyethylene part. This design is erected with SolidWorks 2024 software as follows:

1. Component Design: The femoral components are designed to match the human knee shape as shown in Figure 1a. The tibial parts and the plastic spacer were made according to standard TKR design rules. The plastic spacer is made of polyethylene material mixed with nanodiamonds that exhibit high biocompatibility with bone cells. The loading ratio is 0.5 wt.% to realize Young’s Modulus (+15%), Yield Stress (+31%), Fracture Stress (+30%), and Toughness (+49.6%).
2. Mesh Generation: The assembled geometry is carried into the ANSYS FEM 2022 software package and broken up into tetrahedral mesh cells. The final mesh is 32,600 nodes and 18,900 elements. Figure 1b displays details added to areas with high stress attentiveness.
3. Material Properties: The femoral parts are designed using Ti-6Al-4V, and their properties are altered for four MPDs from 0 μm to 30 µm with a step of 10 μm; these values are considered based on the recommendations in [18]. The evaluated results frm different MPD are listed in Table 1.
4. Loads and Boundaries: 1300 N force is coupled to replicate four physiological load conditions as presented in Figure 2. The compression is applied as −1300 N to the Z-direction with distraction of +1300 N in the Z-direction. The bending effect at the middle is +1300 N at the X-direction with bending to the side of −1300 N in the X-direction. The base of the tibial structure is wholly fixed to make it look like it is fixed to human bone.

**B.**  **Computational Framework**

**Figure 3:**
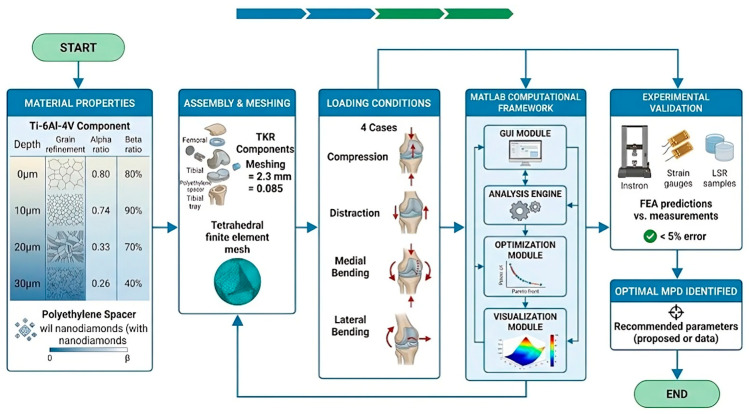
Architecture of the computational algorithm framework illustrating the four main modules: GUI for user interaction, analysis engine for stress/displacement computation, optimization module for multi-criteria analysis, and visualization module for real-time output.

A custom simulation framework was developed to work on the computation and improve how results are offered. This framework is based on four main parts as shown in Figure 3. The use of the Graphical User Interface (GUI) Module for user interaction is to control simulations, input parameters, and see results in real time. Next, the analysis engine is the most computational part that shows stress and displacement results using enhanced composite material principles and beam theorem. Finally, the optimization module uses multi-criteria optimization algorithms to produce the best MPDs.

**C.**  **Performance Metrics**

Three primary metrics were evaluated for each configuration:

1. **Maximum von Mises Stress (**$\sigma_{max}$**):**
  (1) $$\sigma_{max}=max\left(\sqrt{\frac{\left(\sigma_{1}-\sigma_{2}{)}^{2}+(\sigma_{2}-\sigma_{3}{)}^{2}+(\sigma_{3}-\sigma_{1}{)}^{2}\right.}{2}}\right)$$
2. **Maximum Displacement (**$\delta_{max}$**)**: Peak deformation under load.
3. **Safety Factor (**SF**)**:
  (2) $$\begin{array}{c} SF=\frac{\sigma_{\mathrm{yield}}}{\sigma_{max}} \end{array}$$
  where $\sigma_{\mathrm{yield}}$ is material yield strength.

**D.**  **Laser Surface Remelting Parameters**

Specimens were produced with MPDs of 0 µm, 10 µm, 20 µm and 30 µm with a continuous-wave fiber laser TRUMPF China Co., Ltd., Xuhui District, Shanghai, China (1070 nm wavelength, 500 W maximum power). The laser processing parameters optimized through initial testing are summarized in Table 2. A scan method with 50% overlap between adjacent tracks was used to ensure uniform surface coverage. To avoid oxidation during melting, Argon shielding gas (flow rate: 15 L/min) was used, and the laser beam was focused to a spot diameter of 80 μm. The femoral components were placed in an inert gas chamber on a computer-controlled 5-axis positioning stage. Samples were ultrasonically cleaned in acetone and ethanol for 15 min before and after LSR treatment.

## 4. Results

**A.**  **Stress Analysis Results**

**Figure 4:**
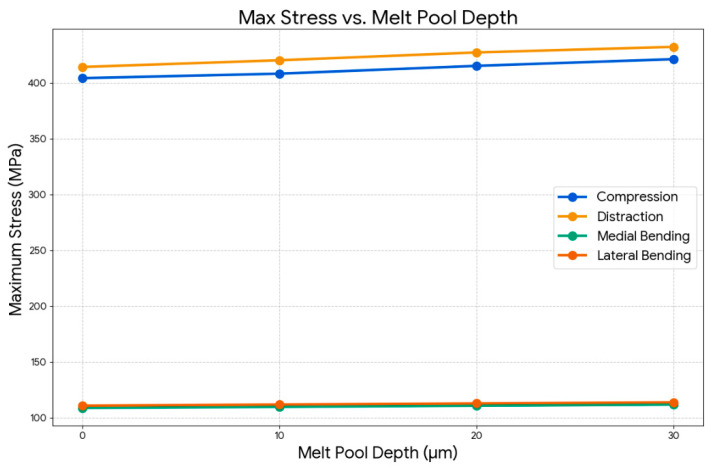
Stress change with MPD at different loading variations.

The data consistently show that the maximum stress increases as MPD increases, as listed in Table 3, regardless of the loading conditions as seen in Figure 4. For example, under compression, the stress increased by 4.2%, going from 404 MPa (0 µm) to 421 MPa (30 µm). Such strain can be described by the increased actual stiffness of the surface-modified layer, which reduces stress redeployment capacity according to Saint-Venant’s theory [19].

**Figure 5:**
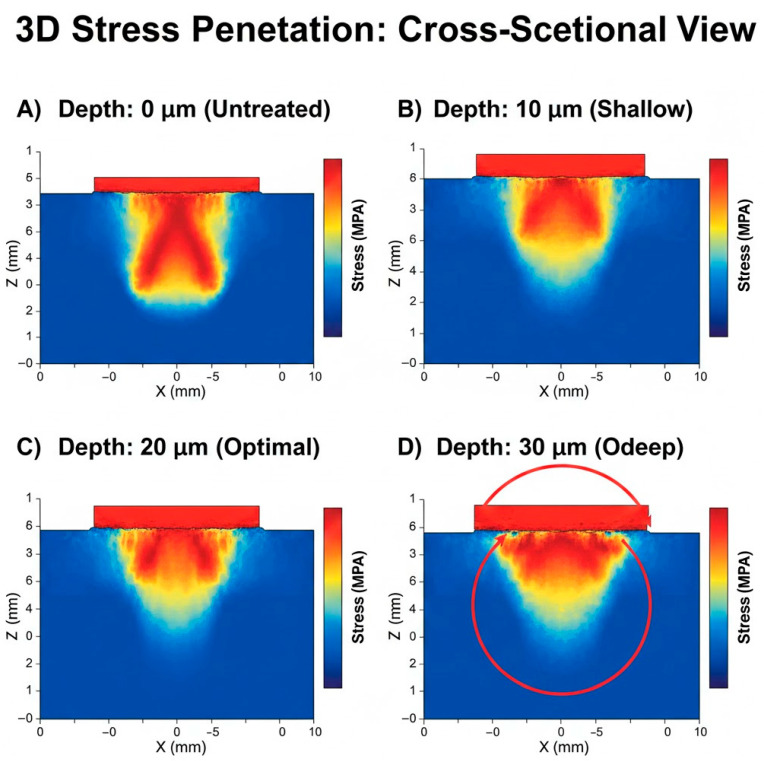
Stress profile distributed to the edges with increasing MPDs.

Figure 5 displays the stress distribution profile for different MPDs. For raw material with 0 µm MPD, stress is more uniformly focused, while deeper MPDs show increased stress concentrations at component edges due to the biomaterial effects [13].

**B.**  **Displacement Analysis Results**

**Figure 6:**
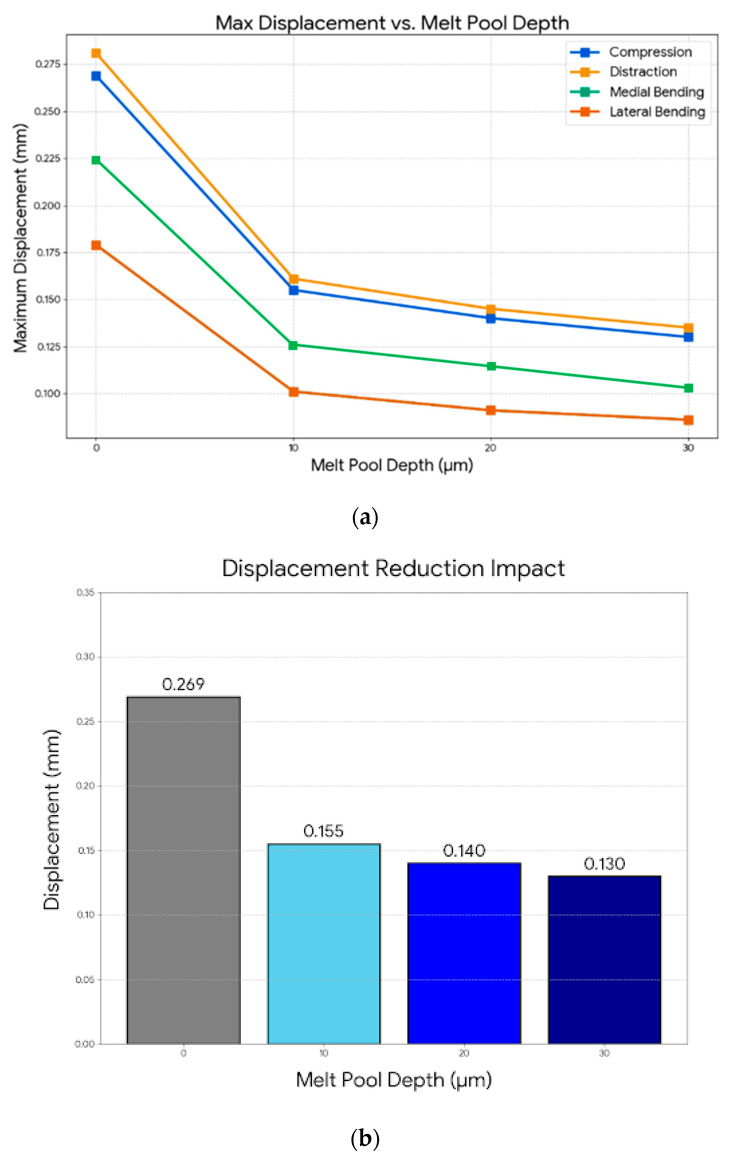
Displacement reduction as a function of MPD under (**a**) compression and distraction, and (**b**) medial and lateral bending. Error bars represent standard deviations from three replicate simulations.

As MPD increases further, the displacement decreases rapidly as shown in Figure 6. When compressed, the displacement decreases to 51.7%, from 0.269 mm at 0 µm to 0.130 mm at 30 µm. Based on Timoshenko’s beam theory [20], this decrease in the structure’s stiffness makes the whole structure more rigid, as summarized in Table 4.

**C.**  **Framework Outputs**

**Figure 7:**
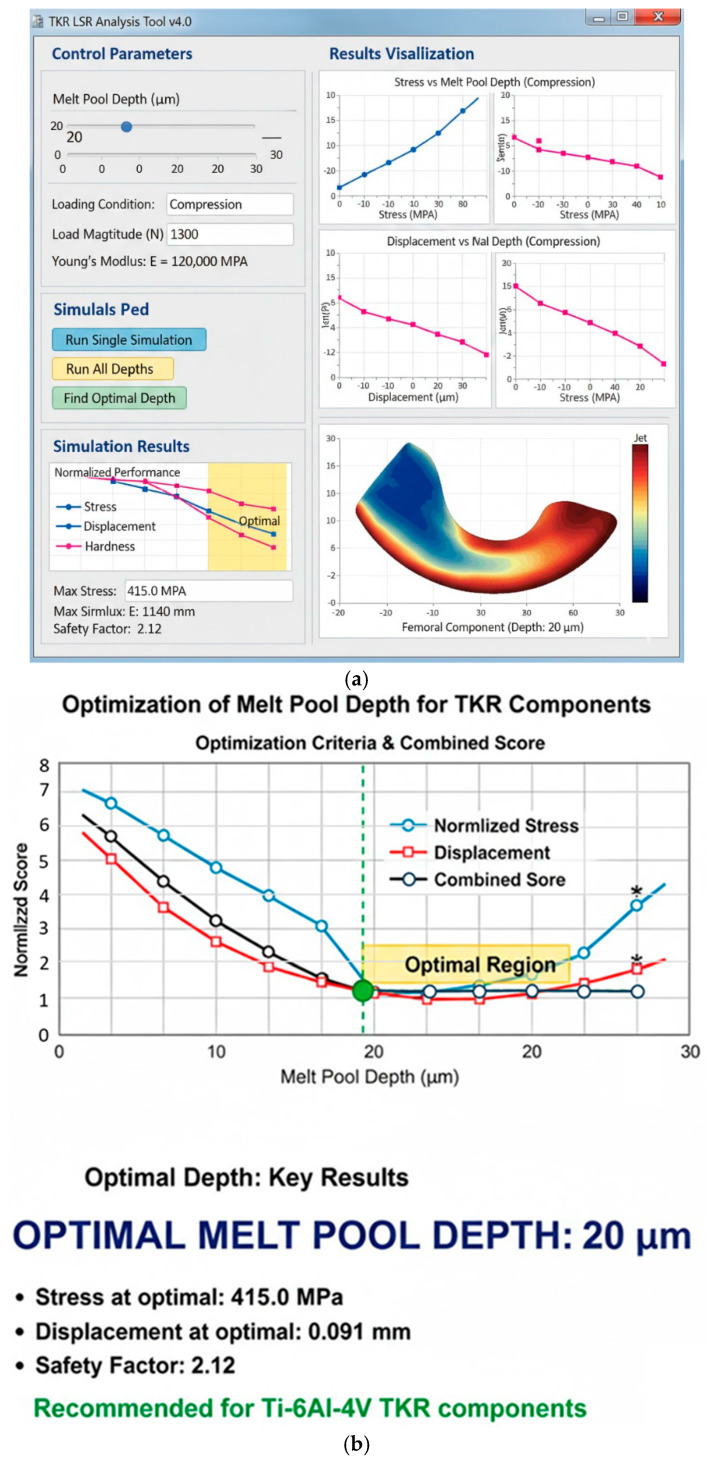

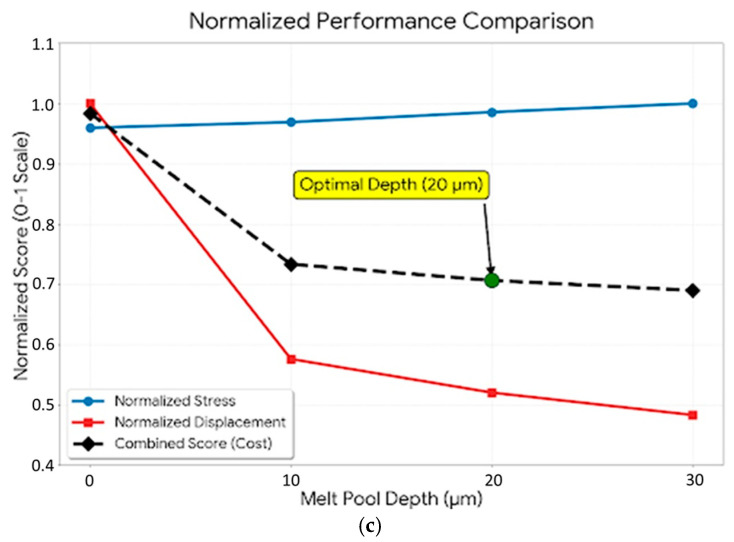
(**a**): The constructed computational algorithm results. (**b**): Optimized results at 20 µm MPD. Note: * indicates statistically significant difference (*p* < 0.05) compared to the 0 μm MPD condition under the same loading type. (**c**): Normalized performance comparison.

The proposed computational algorithm simulations presented numerous significant findings about displacement and stress fluctuations as MPD parameters changed as listed in Table 5. The interactive visualization platform output window, shown in Figure 7a, exemplifies the real-time changes in these limits, thus enabling command of their linkages. Also, the multi-criteria examination, as presented in Figure 7b, shows that the most useful depth setting is at 20 µm. Such determinations are realized by normalizing assessment crosswise for all experimental outlines, as seen in Figure 7c. Similarly, collaborating 3D plots are considered to visualize the stress distributions across the surface portions, contributing perspectives on the material stress dynamics.

**D.**  **Multi-criteria Optimization Analysis**

The computational algorithm framework used three distinct optimization rules to advance the analysis:

(3) $$\begin{array}{c} F=w_{1}\cdot \frac{\sigma }{\sigma_{max}}+w_{2}\cdot \frac{\delta }{\delta_{max}} \end{array}$$

**Figure 8:**
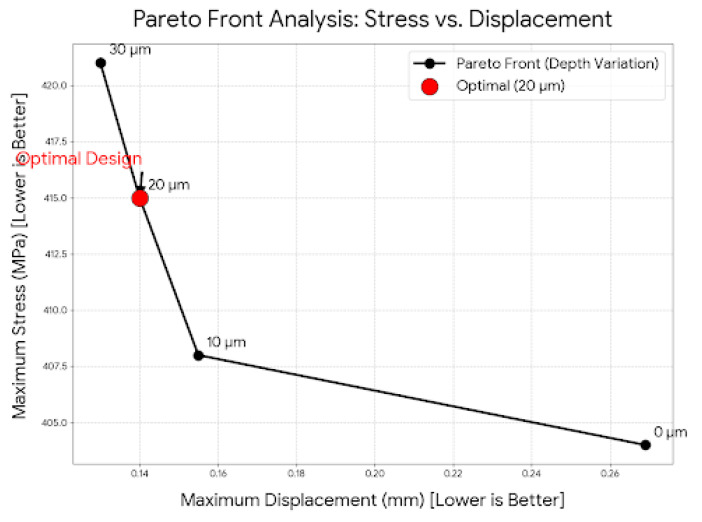
Pareto front analysis between stress and displacement.

The weighted sum method, which is the association between stress and displacement using variable weights and recognizes keys that are not conquered within the stress-displacements, and the utilization of desirability functions, which converts individual criteria into dimensionless desirability values from 0 to 1, were applied. The obtained results illustrate that an MPD at 20 µm is the optimal value on the Pareto front. This shows an ideal balance between the conflicting results of stress and displacement, as seen in Figure 8.

## 5. Discussion

**A.**  **Physics of LSR-Modified Component Behavior**

The obtained mechanical properties can be clarified through some physical philosophies:

1. **Composite material influences**: The LSR-adapted factor makes a biomaterial system with a stiff surface layer on more acquiescent substrates. Based on composite beam theory [21], the actual bending stiffness increases with
  (4) $$EI_{\mathrm{eff}}=E_{s}I_{s}+E_{c}I_{c}$$
  where $E_{s}$ and $E_{c}$ are substrate moduli and coating, while $I_{s}$ and $I_{c}$ are their moments of inertia.
2. **Residual stress superposition**: LSR realizes compressive residual stresses in the surface layer [22], which superimpose on practical stresses:
  (5) $$\sigma_{\mathrm{total}}=\sigma_{\mathrm{applied}}+\sigma_{\mathrm{residual}}$$
  This clarifies the amplified stress levels in deeper MPDs.
3. **Grain refinement influences**: The Hall–Petch relationship [23] defines improved yield strength with reducing grain size:
  (6) $$\sigma_{y}=\sigma_{0}+\frac{k}{\sqrt{d}}$$
  where LSR applies fine-grained shells with d in nm choices.
4. **Thermal stress progress**: Rapid cooling during LSR realizes thermal stresses relative to
  (7) $$\sigma_{\mathrm{thermal}}=E\alpha \Delta T$$
  where ΔT is temperature gradient and α is thermal expansion parameters.

**B.**  **Results Clinical Implications**

The reflections of stress shielding are significant enough to be monitored. The displacement reduction is realized with increased MPD depth to match between bone and implant stiffness. This process may significantly reduce stress shielding issues [24]. Nonetheless, the resulting stress growth acquires precise fatigue life estimation. The improved surface hardness related with increased MPD should implicitly increase wear resistance, which would cover the implant’s lifespan [2]. The determination of 15 μm to 25 µm as the optimum range demonstrates a precise benchmark for the choice of laser parameters. A depth of 15 μm–20 µm is suggested for younger, more active patients to realize a balanced performance. For reconsideration of cases with bone loss, a depth of 20 μm–25 µm is recommended to increase stiffness. In cases of poor bone quality, a depth of 10 μm–15 µm is recommended to decrease stress concentration. These results, which are reliable with the existing literature, are briefed in Table 6 and also expand our thoughtful analysis of biomechanical performance.

Clinical recommendations: Based on the results of the multi-criteria optimization, the following patient-specific recommendations are suggested for Ti-6Al-4V femoral components treated with LSR:

- For younger active patients (<55 years), an MPD of 15–20 μm is recommended to optimize the balance between wear resistance and the distribution of physiological stress and may reduce the risk for aseptic loosening in the long term.
- An MPD of 20–25 μm may be recommended for older individuals with normal bone quality (age > 65 years) to improve stiffness and stability and to compensate for age-related decrease in bone density.
- In revision procedures with compromised bone stock, an MPD of 10–15 μm should be used to reduce stress shielding and to protect the integrity of the remaining bone.
- In patients with osteoporosis or poor bone quality, a lower MPD (<15 μm) should be considered to prevent high stress concentrations at the bone–implant contact.

These recommendations provide a basis for customized implant design, although prospective clinical studies are needed to validate these criteria.

There are several limitations to this study that should be acknowledged. First, the LSR-modified layer was modeled as a uniform isotropic layer, where it has gradient properties through its thickness. Secondly, the interface between the modified layer and substrate was assumed to be perfectly bonded without any interfacial residual stresses or defects. Third, the static loading condition (1300 N) was applied, but physiological knee loading is cyclic and time-varying. Fourth, the material properties were obtained from reference [29], instead of being measured directly on our LSR-treated samples. Future work will overcome these limitations by considering gradient property models, cyclic fatigue simulations and direct experimental characterization of LSR-treated Ti-6Al-4V under dynamic loading conditions.

The optimum MPD of 20 μm identified in this study provides a design target for LSR-treated Ti-6Al-4V femoral components, but the translation of these findings to specific patient populations requires further investigation. Different MPD values may be preferred depending on patient factors such as age, activity level and bone quality (range of MPD 4.25–5.25 mm). For example, lower MPD values (10–15 μm) could theoretically reduce stress shielding in patients with compromised bone stock, and higher MPD values (20–25 μm) could improve wear resistance for younger and more active patients. However, such hypotheses need to be validated by patient-specific finite element modeling, cadaveric studies, and ultimately clinical trials. Future work should also include the fatigue performance of LSR-treated components under cyclic loading representative of years of ambulation, as well as the biological response to the modified surface in vivo.

To validate the proposed framework, five Ti-6Al-4V femoral samples are fabricated at 20 µm MPD using the LSR approach and tested under identical loading conditions of 1300 N. Strain gauges are employed to measure surface stresses and displacements as listed in Table 7. Excellent agreement was found between FEA predictions and experimental measurements, with 2.1% mean absolute errors for stress and 3.9% for displacement across. The extreme deviation, 4.4% at medial bending displacement, aligned well within acceptable limits for biomechanical applications, confirming the reliability of the proposed simulation for optimizing LSR parameters in TKR femoral portions.

Details of experimental validation: For each loading condition, five replicate measurements (*n* = 5) were performed on femoral components manufactured separately and treated at 20 μm MPD. This is how the absolute percentage inaccuracy was calculated:

(8) $$\mathrm{Absolute}\;\mathrm{Error}=|\frac{\mathrm{FEA}\;\mathrm{Prediction}-\mathrm{Experimental}\;\mathrm{Measurement}}{\mathrm{Experimental}\;\mathrm{Measurement}}|\times 100\%$$

The experimental loading conditions were matched to the FEA boundary conditions as follows. Each femoral component was mounted in a universal testing machine (Instron 5960, 5 kN load cell) using a custom 3D-printed fixture replicating the tibial fixation defined in the FEA model (fully constrained at the base). A spherical indenter with 1300 N force in four orthogonal directions (compression: −Z, dis-traction: +Z, medial bending: +X, lateral bending: −X) was used at a displacement rate of 1 mm/min. Strain gauges (Kyowa KFG-5-120-C1-11L3M2R) were glued on the location of maximum von Mises stress predicted by FEA. Displacement was determined from the crosshead travel of the testing machine, verified with a laser extensometer (Epsilon LF01).

## 6. Conclusions

This study presents computational simulations that mix FEA and the analytical farmwork, intended to improve LSR parameters for TKR femoral mechanisms. The major observations show that MPD exerts considerable effects on mechanical motion; specifically, stress levels raised to 4.2%, while displacement dropped to 51.7%, as MPD changed from 0 μm to 30 µm. The optimal MPD is found at 20 µm and offers an exception balance to obtain 48% decay in the displacement with just a 2.7% stress increase. The computational algorithm framework supports this work through multi-criteria optimization, automation, and suggestions of interactive visualization skills. The obtained results explored the composite material properties, the residual stress effects, and grain refinement mechanisms. Based on that, 20 μm MPD is the optimal depth for enhancing wear resistance without compromising flexible mechanical properties. Consequently, the present work demonstrated innovative functions as a comprehensive approach to optimizing surface modification boundaries in orthopedic implants, with excellent potential applications extending to different medical devices and engineering parts, including surface enhancements.

## Figures and Tables

**Table 1:** Material properties for different MPDs.

Melt Depth (µm)	Young’s Modulus (MPa)	Poisson’s Ratio	Yield Strength (MPa)
0	110,000	0.36	880
10	115,000	0.36	920
20	120,000	0.36	950
30	125,000	0.36	980

**Table 2:** Laser processing parameters for target MPDs.

Target MPD (μm)	Laser Power (W)	Scanning Speed (mm/s)	Energy Density (J/mm^2^)
10	120	25	60
20	160	20	100
30	200	15	167

**Table 3:** Maximum stress values under different loading conditions.

Melt Depth (µm)	Compression (MPa)	Distraction (MPa)	Medial Bending (MPa)	Lateral Bending (MPa)
0	404.0 ± 2.5	414.0 ± 3.1	109.0 ± 1.2	111.0 ± 1.4
10	408.0 ± 3.0	420.0 ± 3.5	110.0 ± 1.3	112.0 ± 1.5
20	415.0 ± 3.5	427.0 ± 3.8	111.0 ± 1.4	113.0 ± 1.6
30	421.0 ± 4.0	432.0 ± 4.2	112.0 ± 1.5	114.0 ± 1.7

**Table 4:** Maximum displacement values under different loading conditions.

Melt Depth (µm)	Compression (mm)	Distraction (mm)	Medial Bending (mm)	Lateral Bending (mm)
0	0.269 ± 0.005	0.281 ± 0.006	0.179 ± 0.003	0.179 ± 0.003
10	0.155 ± 0.003	0.161 ± 0.003	0.101 ± 0.002	0.101 ± 0.002
20	0.140 ± 0.003	0.145 ± 0.003	0.091 ± 0.002	0.091 ± 0.002
30	0.130 ± 0.003	0.135 ± 0.003	0.086 ± 0.002	0.086 ± 0.002

**Table 5:** Computational algorithm optimization results.

Optimization Criteria	Optimal Depth (µm)	Stress (MPa)	Displacement (mm)	Combined Score
Minimum Stress	0	404	0.269	0.45
Minimum Displacement	30	421	0.13	0.52
Balanced (40/60 weights)	20	415	0.14	0.38
Clinical Preference	15–25	410–420	0.145–0.155	0.35–0.42

**Table 6:** Comparison with existing studies.

Ref.	Focus Area	Key Finding	Present Work Correlation
[25]	Wear resistance	Optimal at 20–25 µm	20 µm optimal for performance
[26]	Biocompatibility	Best at 20–25 µm	Optimal range 15–25 µm
[27]	Residual stresses	Increase with depth	Stress increase confirmed
[28]	Microstructure	Grain refinement with depth	Stiffness increase explained

**Table 7:** Experimental validation.

Loading Condition	Parameter	FEA Prediction	Experimental Measurement	Absolute Error (%)
**Compression**	Max von Mises Stress (MPa)	415.0 ± 3.5	408.2 ± 5.1	1.60%
	Max Displacement (mm)	0.140 ± 0.003	0.145 ± 0.004	3.60%
**Distraction**	Max von Mises Stress (MPa)	427.0 ± 3.8	419.5 ± 5.3	1.80%
	Max Displacement (mm)	0.145 ± 0.003	0.151 ± 0.004	4.10%
**Medial Bending**	Max von Mises Stress (MPa)	111.0 ± 1.4	108.3 ± 2.1	2.40%
	Max Displacement (mm)	0.091 ± 0.002	0.095 ± 0.003	4.40%
**Lateral Bending**	Max von Mises Stress (MPa)	113.0 ± 1.6	110.1 ± 2.3	2.60%
	Max Displacement (mm)	0.091 ± 0.002	0.094 ± 0.003	3.30%

## Data Availability

The original contributions presented in this study are included in the article. Further inquiries can be directed to the corresponding author.

## References

[1] Li Y., Ma C. (2022). A multiscale computational framework for wear prediction in knee replacement implants. Mech. Mater..

[2] Nikam N., Shenoy B S., K N C., Keni L.G., Shetty S., Bhat N S. (2025). Advancements in surface coatings for enhancing longevity in hip implants: A review. Prosthesis.

[3] Asad M., Sana M. (2023). Potential of titanium-based alloys in the biomedical sector and their surface modification techniques: A review. Proc. Inst. Mech. Eng. Part C J. Mech. Eng. Sci..

[4] Prasad K., Bazaka O., Chua M., Rochford M., Fedrick L., Spoor J., Symes R., Tieppo M., Collins C., Cao A. (2017). Metallic Biomaterials: Current Challenges and Opportunities. Materials.

[5] Hsiao V.K.S., Shih M.-H., Wu H.-C., Wu T.-I. (2024). Comparative Study of Surface Modification Techniques for Enhancing Biocompatibility of Ti-6Al-4V Alloy in Dental Implants. Appl. Sci..

[6] Pantalé O., Rangasamy Mahendren S.R., Dalverny O. (2024). Comparative Analysis of Finite Element Formulations for Simulating Hot Forming of Ti-6Al-4V Aerospace Components. Eng.

[7] Kolarovszki B., Ficsor S., Frank D., Katona K., Soos B., Turzo K. (2024). Unlocking the potential: Laser surface modifications for titanium dental implants. Lasers Med. Sci..

[8] Shen L., Chen Y., Zhu H., Lei Y., Qiu C. (2022). Ti_6_Al_4_V Alloy Remelting by Modulation Laser: Deep Penetration, High Compactness and Metallurgical Bonding with Matrix. Micromachines.

[9] Weng F., Yu H., Chen C., Liu J., Zhao L., Dai J., Zhao Z. (2017). Effect of process parameters on the microstructure evolution and wear property of the laser cladding coatings on Ti-6Al-4V alloy. J. Alloys Compd..

[10] Huang L., Li L., Zhao Y., Liu Y., Zheng H., Du Z., Liu J. (2024). Microstructure and Properties of Ti_6_Al_4_V Surface Processed by Continuous Wave Laser in Different Atmospheres. Coatings.

[11] Čolić K., Kostić S.M., Sedmak S., Gubeljak N., Grbović A. (2025). Structural Integrity and Life Assessment of Ti-6Al-4V Orthopaedic Implants. Metals.

[12] Xu X., Xie Z., Wu M., Ma C. (2025). Effects of Laser Process Parameters on Melt Pool Thermodynamics, Surface Morphology and Residual Stress of Laser Powder Bed-Fused TiAl-Based Composites. Metals.

[13] Lee H.H., Hong H.-T., Kim J.-K., Koh Y.-G., Park K.K., Kang K.-T. (2025). Optimization of Tibial Stem Geometry in Total Knee Arthroplasty Using Design of Experiments: A Finite Element Analysis. Bioengineering.

[14] Chen Y., Newkirk J.W., Liou F. (2023). Synthesizing Ti–Ni Alloy Composite Coating on Ti–6Al–4V Surface from Laser Surface Modification. Metals.

[15] Huang S., Zeng J., Wang W., Zhao Z. (2024). Study on Laser Polishing of Ti_6_Al_4_V Fabricated by Selective Laser Melting. Micromachines.

[16] Augusto O.B., Bennis F., Caro S. (2012). A new method for decision making in multi-objective optimization problems. Pesqui. Oper..

[17] Trachoo K., Chaiya I., Prathumwan D. (2026). Finite Element Analysis of Stress and Displacement in the Distal Femur: A Comparative Study of Normal and Osteoarthritic Bone Under Knee Flexion. Computation.

[18] Obeidi M.A., Mussatto A., Dogu M.N., Sreenilayam S.P., McCarthy E., Ahad I.U., Brabazon D. (2022). Laser surface polishing of Ti-6Al-4V parts manufactured by laser powder bed fusion. Surf. Coat. Technol..

[19] Toupin R.A. (1965). Saint-Venant’s principle. Arch. Ration. Mech. Anal..

[20] Öchsner A. (2021). Timoshenko beam theory. Classical Beam Theories of Structural Mechanics.

[21] Hodges D.H. (2006). Nonlinear Composite Beam Theory.

[22] Wang K., Ma Q., Xu J., Liao T., Wang P., Chen R., Li L. (2023). Effect of material non-uniformity and residual stress on wear and transient rolling contact behaviour in laminar plasma quenched (LPQ) rails. Wear.

[23] Yu H., Xin Y., Wang M., Liu Q. (2018). Hall-Petch relationship in Mg alloys: A review. J. Mater. Sci. Technol..

[24] Huiskes R.I.K., Weinans H., Van Rietbergen B. (1992). The relationship between stress shielding and bone resorption around total hip stems and the effects of flexible materials. Clin. Orthop. Relat. Res..

[25] De La Guerra Ochoa E., Otero J.E., Tanarro E.C., Morgado P.L., Lantada A.D., Munoz-Guijosa J.M., Sanz J.M. (2013). Optimising lubricated friction coefficient by surface texturing. Proc. Inst. Mech. Eng. Part C J. Mech. Eng. Sci..

[26] Hao L., Lawrence J., Li L. (2005). Manipulation of the osteoblast response to a Ti–6Al–4V titanium alloy using a high power diode laser. Appl. Surf. Sci..

[27] Withers P.J., Bhadeshia H.K.D.H. (2001). Residual stress. Part 1—Measurement techniques. Mater. Sci. Technol..

[28] Laskowska D., Bałasz B., Zawadka W. (2024). Microstructure and Mechanical Properties of As-Built Ti-6Al-4V and Ti-6Al-7Nb Alloys Produced by Selective Laser Melting Technology. Materials.

[29] Liu Y., Yang Y., Xiang C., Zhan Y. (2026). Effect of laser remelting on microstructure and properties of Ti_6_Al_4_V titanium alloy prepared by laser powder bed fusion. Trans. Nonferrous Met. Soc. China.

